# Ratiometric nonfluorescent CRISPR assay utilizing Cas12a-induced plasmid supercoil relaxation

**DOI:** 10.1038/s42004-024-01214-2

**Published:** 2024-06-08

**Authors:** Noor Mohammad, Logan Talton, Selen Dalgan, Zach Hetzler, Anastasiia Steksova, Qingshan Wei

**Affiliations:** 1 Department of Chemical and Biomolecular Engineering, North Carolina State University, Raleigh, NC 27695 USA; 2 Department of Chemical Engineering, Bangladesh University of Engineering and Technology, Dhaka, 1000 Bangladesh

**Keywords:** DNA, Sensors, Bioanalytical chemistry

## Abstract

Most CRISPR-based biosensors rely on labeled reporter molecules and expensive equipment for signal readout. A recent approach quantifies analyte concentration by sizing λ DNA reporters via gel electrophoresis, providing a simple solution for label-free detection. Here, we report an alternative strategy for label-free CRISPR-Cas12a, which relies on Cas12a *trans*-nicking induced supercoil relaxation of dsDNA plasmid reporters to generate a robust and ratiometric readout. The ratiometric CRISPR (rCRISPR) measures the relative percentage of supercoiled plasmid DNA to the relaxed circular DNA by gel electrophoresis for more accurate target concentration quantification. This simple method is two orders of magnitude more sensitive than the typical fluorescent reporter. This self-referenced strategy solves the potential application limitations of previously demonstrated DNA sizing-based CRISPR-Dx without compromising the sensitivity. Finally, we demonstrated the applicability of rCRISPR for detecting various model DNA targets such as HPV 16 and real AAV samples, highlighting its feasibility for point-of-care CRISPR-Dx applications.

## Abstract (web summary)

CRISPR-Cas proteins appear not only as a genome editing tool but also as a promising diagnostic tool to detect nucleic acid targets, however, most of the CRISPR-based biosensors rely on labelled reporters for signal acquisition. Here, the authors develop a label-free CRISPR-Cas12a-based sensing platform by using supercoiled plasmid DNA as ratiometric reporters, detecting ssDNA targets with a limit of detection of around 100 fM, and applying it to detect various virus samples.

## Introduction

Rapid, inexpensive, and sensitive nucleic acid detection methods are desired for early and point-of-care (POC) diagnostics. Currently, widely applied nucleic acid detection methods, including polymerase chain reaction (PCR) assays^1,2^ and DNA sequencing^3^, are time-consuming, inaccessible for field use, and dependent on expensive instruments and skilled technical staff^4^. Besides, the current gold standard nucleic acid amplification method, PCR, faces trouble detecting small nucleic acid biomarkers such as microRNA (miRNA), as the primer design of PCR makes it infeasible to bind short nucleic acid targets (20 nts or shorter)^5^.

CRISPR (Clustered Regularly Interspaced Short Palindromic Repeats) with associated protein (aka CRISPR-Cas) initially appeared as a genome editing tool^6–9^ and now has evolved into a promising diagnostic tool^10–14^. For diagnostic purposes, CRISPR could be a viable alternative to PCR or DNA sequencing because CRISPR-based diagnostics (CRISPR-Dx) is isothermal, fast, sensitive, specific, and simple^15^. CRISPR-Dx came into the limelight after the discovery of Cas13a-based Specific High-Sensitivity Enzymatic Reporter UnLOCKing (SHERLOCK) platform for detecting RNA sequences^11^, and Cas12a-based DNA endonuclease-targeted CRISPR *trans* reporter (DETECTR) mechanism to detect DNA targets^10^. Numerous CRISPR-Dx platforms have been developed to date, showcasing the immense potential of CRISPR systems in addressing the longstanding challenge of achieving highly sensitive and specific nucleic acid detection through a comparatively straightforward process^12,13,16–48^. CRISPR-Cas12a particularly draws extensive attention, given its ease of reconfiguration for detecting a wide range of human, animal, and plant diseases^10,17,20,23,49–53^, including gene mutations^32,54,55^.

For signal acquisition purposes, CRISPR-Cas12a-Dx typically relies on fluorescent, colorimetric, or electrochemical readout^10,11,16,21,27,29,31,32,34,39,45,50,56–59^. The signal is mainly achieved when CRISPR-Cas12a nonspecifically cleaves the reporter molecules (typically ssDNA) via its unique *trans*-cleavage mode and changes the fluorescence^10,11,16,21,29,31,32,39,45,58^, color^34,50,56,57^, or current states^27,59^ of the reporters. The *trans*-cleavage process can also be monitored by other techniques, such as the surface plasmon resonance (SPR), where AuNP-conjugated ssDNA was used as the reporter^60,61^. One of the major limitations of these signal generation strategies is the need for expensive labels and readout instruments. Instead, we previously demonstrated a simple, inexpensive, and nonoptical CRISPR-Cas12a-based sensing platform to detect short ssDNA at sub-picomolar (pM) detection sensitivity using ds λ DNA as an alternative reporter. This sizing-based method measures the length reduction of λ DNA reporters via gel electrophoresis to quantify target concentrations without any fluorescent labels or complicated optical instrument^15^. However, precise determination of band shift for long DNA reporters (e.g., λ DNA) may be challenging due to the need for including a control λ DNA reporter running on the same gel to serve as the reference. The DNA band positions may also be subject to various interferences, such as gel uniformity and uneven electric field. In addition, the precise band shift of long DNA (e.g., λ DNA) is more difficult to evaluate, as large DNA fragments (>20 kb) are difficult to separate and run nearly at the same speed in the conventional gel electrophoresis technique^62^. Specialized gel electrophoresis (e.g., pulse field gel electrophoresis) is more efficient in separating larger DNA fragments, but is more expensive and complex^62,63^. Recently, the sizing-based detection concept has also been applied to a new class of hybrid reporters (i.e., dsDNA backbone plus a 3’ toe)^64^. Although the separation of short hybrid reporters is much faster and easier, the interference from band distortion still remains.

Here, we demonstrated a self-calibrated and label-free CRISPR-Cas12a-based sensing platform to detect short ssDNA or dsDNA utilizing the *trans*-nicking behavior of Cas12a and conformation change of supercoiled plasmid DNA reporters to generate ratiometric signal output without the need for additional molecular reference. Different from the previous sizing approach that relies on precise band position determination, this new methodology produces ratiometric signals based on the monitoring of relative population of supercoiled versus relaxed circular DNA via band intensity analysis, eliminating many gel distortion-induced interferences. Three circular dsDNA plasmids, namely pUC19 (2.69 kb)^65^, pBR322 (4.36 kb)^66^, and ΦX174 (5.37 kb)^67^ were employed as ratiometric reporters for the rCRISPR assay. The target-activated Cas12a nicks and relaxes supercoiled plasmids via its *trans*-cleavage mode, producing first circular and then linear forms of plasmids. The extent of conformation change (supercoil to circular) induced by *trans*-active Cas12a is proportional to the target concentration. With rCRISPR, we achieved ultrasensitive detection of ssDNA targets with a limit of detection (LOD) of ~100 fM, superior to fluorescent reporter-based systems by around two orders of magnitude. Furthermore, we demonstrated the application of this self-referenced approach for detecting various DNA targets such as human papillomavirus 16 (HPV16) biomarkers and real adeno-associated virus (AAV) samples.

## Results and discussion

### Discovery of the dsDNA plasmid reporter

**Fig. 1:**
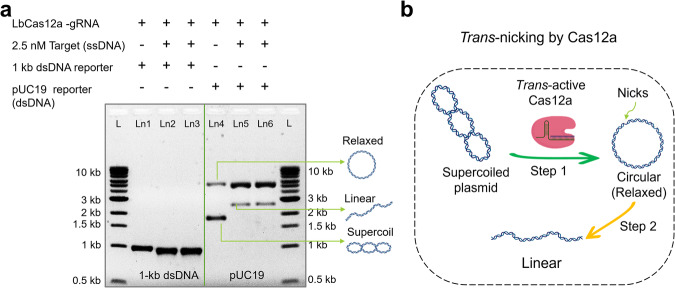
dsDNA plasmid as the CRISPR-Cas12a reporter. **a** Gel electrophoresis (1% agarose gel and 1× TBE buffer) results demonstrating the different CRISPR-Cas12a assay results of using 1-kb linear dsDNA and 2.7 kb supercoiled pUC19 dsDNA plasmid as nonspecific substrates. **b** Cartoon illustration showing the conformation change of pUC19 induced by *trans*-active Cas12a. The supercoiled-circular transition step is utilized for developing a ratiometric CRISPR (rCRISPR) assay later. Abbreviations: gRNA guide RNA, nM nanomolar, ssDNA single-stranded DNA, kb kilobase pair, L 1-kb ladder, Ln lane, TBE Tris-borate EDTA, dsDNA double-stranded DNA.

CRISPR-Cas12a-based diagnostic schemes have mainly been demonstrated based on the *trans*-cleavage of ssDNA reporter by Cas12a protein^10,13,22,29,34,39^. Recently, we discovered that CRISPR-Cas12a can also actively *trans*-cleave ds λ DNA substrate^15^. Although it is still unclear how ds λ DNA gets *trans*-cleaved, the use of dsDNA reporters would not only enrich the varieties of CRISPR-Dx, but it could also overcome the limitations of the typical fluorescence-based reporting system, such as the need for fluorescent labeling and readout. To further explore the *trans*-cleavage behavior of Cas12a on other dsDNA substrates, we chose to screen a set of plasmid DNA molecules. Plasmids are small, circular, dsDNA molecules that can be tightly supercoiled in order to fit inside the cell. In the initial test, two model reporters: 1-kilobase pair (kb) linear dsDNA and 2.69-kb pUC19 dsDNA plasmid, were chosen and compared. To run CRISPR-Cas12a assay, a 20-nt long synthetic ssDNA was used as the artificial target (see sequence in Supplementary Table 1). In Fig. 1a, lanes 1 and 4 are negative controls, where 1-kb dsDNA reporter and pUC19 DNA were used, respectively, but no target was added. As expected, no reporter cleavage was observed since the target was not there to activate LbCas12a. It is worth mentioning that intact pUC19 plasmid has two distinct bands in lane 4, indicating the presence of supercoiled and relaxed (circular) conformations of pUC19. Lanes 2 and 3 were testing lanes for the 1-kb dsDNA reporter and 2.5 nM ssDNA target was present. Similarly, lanes 5 and 6 were testing lanes for pUC19 DNA reporter in the presence of targets. No collateral cleavage was observed for the 1 kb dsDNA reporter when targets were added (Fig. 1a, lanes 2 and 3), which confirms the conventional knowledge of the field that CRISPR-Cas12a does not typically *trans*-cleave dsDNA substrates (λ DNA is an exception)^10^. However, for the pUC19 reporter, we observed a significant change in band pattern in the presence of target (Fig. 1a, lanes 5 and 6). The supercoiled band (lower band) disappeared, the circular band (upper band) was enhanced in intensity, and finally, a new band appeared in between. We attribute this interesting pattern change to the conformation alternation of the pUC19 reporter, which underwent *supercoiled-circular-linear* transformation due to the enzymatic activity of Cas12a (Fig. 1b). We believe the following steps occur in a sequence: first, *trans*-active Cas12a would preferably nick the twisting knots of supercoiled pUC19 (we call it *trans-*nicking) and relax pUC19 into the lower energy circular form (or relaxed form). Next, excess target-activated Cas12a could further *trans*-degrade circular pUC19 into linear fragments by completely breaking the previous nicking site (Fig. 1b). The nicking effect of activated Cas12a was recognized in a few previous reports^68^, but it is the first time that such nicking behavior can be utilized for plasmid relaxation.

### Nonfluorescent ratiometric CRISPR-Cas12a assay (rCRISPR) utilizing plasmid supercoil relaxation

**Fig. 2:**
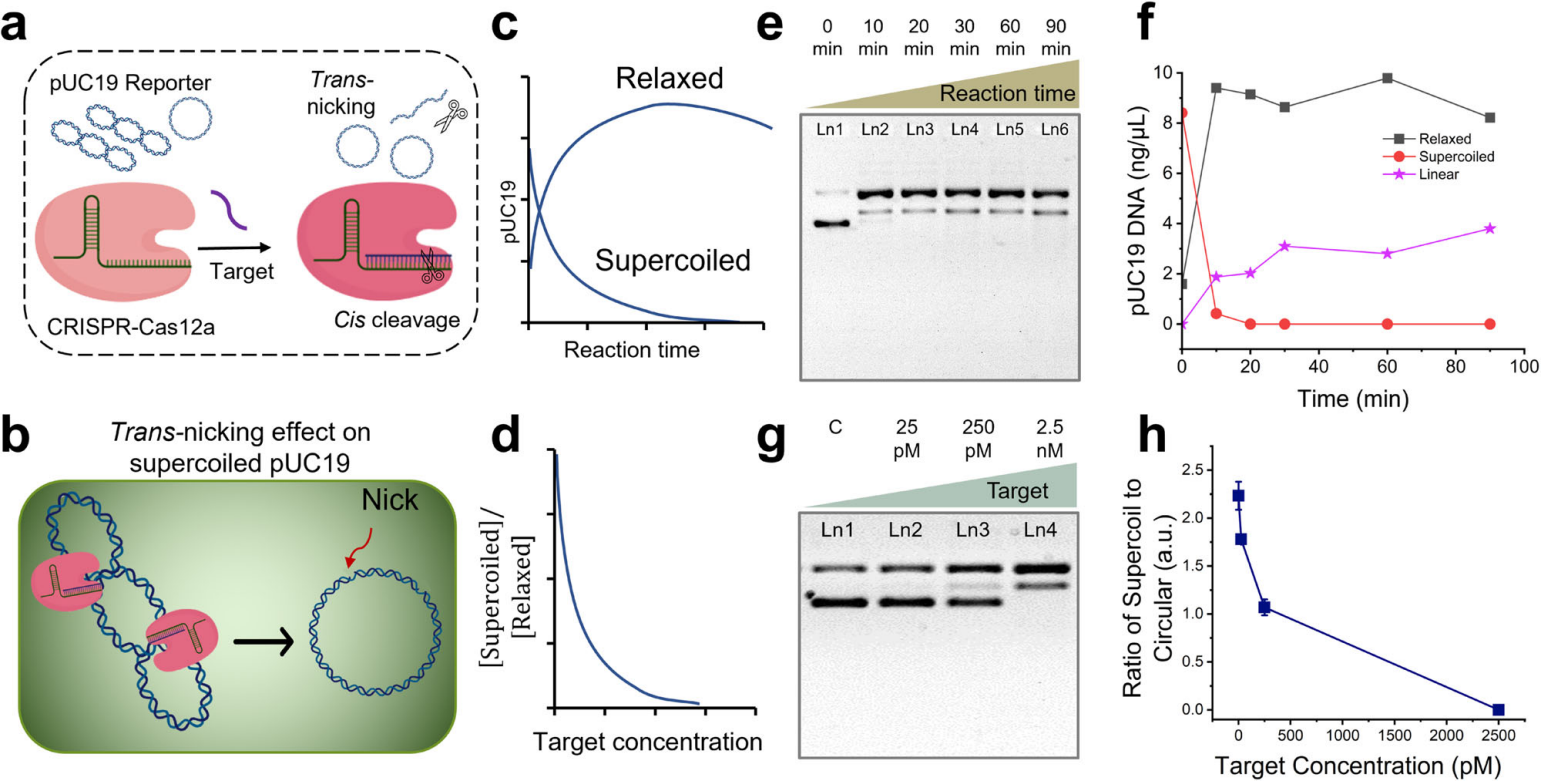
Ratiometric CRISPR-Cas12a assay using plasmid reporters. **a** Schematic of the CRISPR-Cas12a assay using pUC19 as a nonspecific substrate. **b** Close-up cartoon view showing activated Cas12a nicks the supercoil pUC19 and converts it into relaxed conformation. **c** Hypothetical graphical illustration showing how the percentage of supercoil and relaxed DNA would change as the CRISPR reaction proceeds. **d** Hypothetical graphical illustration showing how the ratio of supercoil DNA to relaxed circular DNA could change with the target concentrations. Gel electrophoresis (1% agarose gel and 1× TBE buffer) results (**e**) and scatter plot (**f**) demonstrating the nonspecific nicking of pUC19 by Cas12a over the reaction time course in the presence of 2.5 nM ssDNA target. DNA concentration (ng per μL) was estimated by comparing the band intensities and converted from the initial plasmid DNA concentration. Gel electrophoresis (1% agarose gel and 1× TBE buffer) results (**g**) and scatter plot (**h**) demonstrating the effect of target concentration on *trans*-nicking of pUC19 by Cas12a for a 30-min reaction. Abbreviations: c negative control, pM picomolar, nM nanomolar, TBE Tris-borate EDTA, a.u. arbitrary unit.

Next, we evaluated the feasibility of using the gel pattern change of the plasmid reporter as a self-calibrated sensing mechanism. The developed ratiometric CRISPR assay is termed rCRISPR for simplicity. To demonstrate the nonoptical rCRISPR assay based on dsDNA supercoil relaxation, we ran CRISPR-Cas12a reaction following the standard protocol, except that we used pUC19 dsDNA as the reporter molecule. Figure 2a shows the schematic of rCRISPR assay using pUC19 DNA as the reporter molecule. Figure 2b shows the cartoon schematic illustrating the Cas12a nicking and conversion of supercoiled pUC19 into relaxed circular conformation. Figure 2c, d denote the graphical predictions showing how the fraction of supercoiled and relaxed circular DNA would change as the CRISPR reaction proceeds at a fixed target concentration, and how the ratio of supercoiled DNA to relaxed circular DNA could change with the target concentrations at a fixed reaction time, respectively.

To verify the prediction, we ran rCRISPR reactions with pUC19 DNA reporters for 10 min, 20 min, 30 min, 60 min, and 90 min. We used 2.5 nM ssDNA target to *trans*-activate Cas12a. We observed that within 10 minutes of reaction, all supercoiled DNA vanished (aka. converted to relaxed form) (Fig. 2e). Converting relaxed circular DNA into linear conformation might happen instantly with the supercoil relaxation; however, the linearization process was observed up to 90 min of reaction, as indicated by the slow darkening of the linear form band. Notably, no degradation of linear dsDNA was observed for the given reaction conditions (90 min of reaction, 2.5 nM target) (Fig. 2e). The sharp slope in Fig. 2f for supercoiled DNA confirmed that the DNA supercoil relaxation is very fast compared to the 2^nd^ step (DNA linearization) and potentially 3rd step (degradation of liner DNA, which happens only for high target concentrations). This also could be the reason why we did not see much difference in gel patterns when we tested different assay times for 1 h, 2 h, and 3 h (Supplementary Fig. 1). Since the longer reaction time did not affect the degradation of linear DNA at 2.5 nM target concentration, we performed additional experiments with higher target concentrations (up to 50 nM) and observed full degradation of linear pUC19 for 50 nM target after 1 hr reaction (Supplementary Fig. 2). We believe that the *trans*-nicking of supercoil plasmids occurs even faster than *trans*-cleavage of fluorescent reporters. In the case of conventional ss fluorescent reporters, the CRISPR reaction will usually take >15 min to ensure all F-Q reporters are properly cleaved^17^ and generate detectable signals. In contrast, with similar analyte concentration (i.e., 2.5 nM ssDNA), the supercoil relaxation can be completed <10 min (Fig. 2e) and therefore could be advantageous for developing a faster-response CRISPR biosensor.

We then performed the assay with 25 pM, 2.5 nM, 25 nM, and 0 targets (negative control) and evaluated the feasibility of ratiometric sensing for 30 min reaction time (Fig. 2g). We divided the intensity of the supercoiled band (lower band in each lane in Fig. 2g) by the intensity of the relaxed circular band (upper band in each lane in Fig. 2g) to get the ratiometric intensity. We plotted the ratiometric intensity against different target concentrations, and observed that the ratiometric signal decreased as a function of target concentration (Fig. 2h), following a similar trend to the prediction (Fig. 2d). This initial data demonstrates the feasibility of using plasmid DNA as a ratiometric reporter for sensitive nucleic acid detection, which overcomes the limitations of the previously demonstrated DNA-sizing-based sensing^15^ and also excludes the need for expensive dye reporter molecules or sophisticated optical equipment.

### The universality of supercoil relaxation among different dsDNA plasmid reporters

**Fig. 3:**
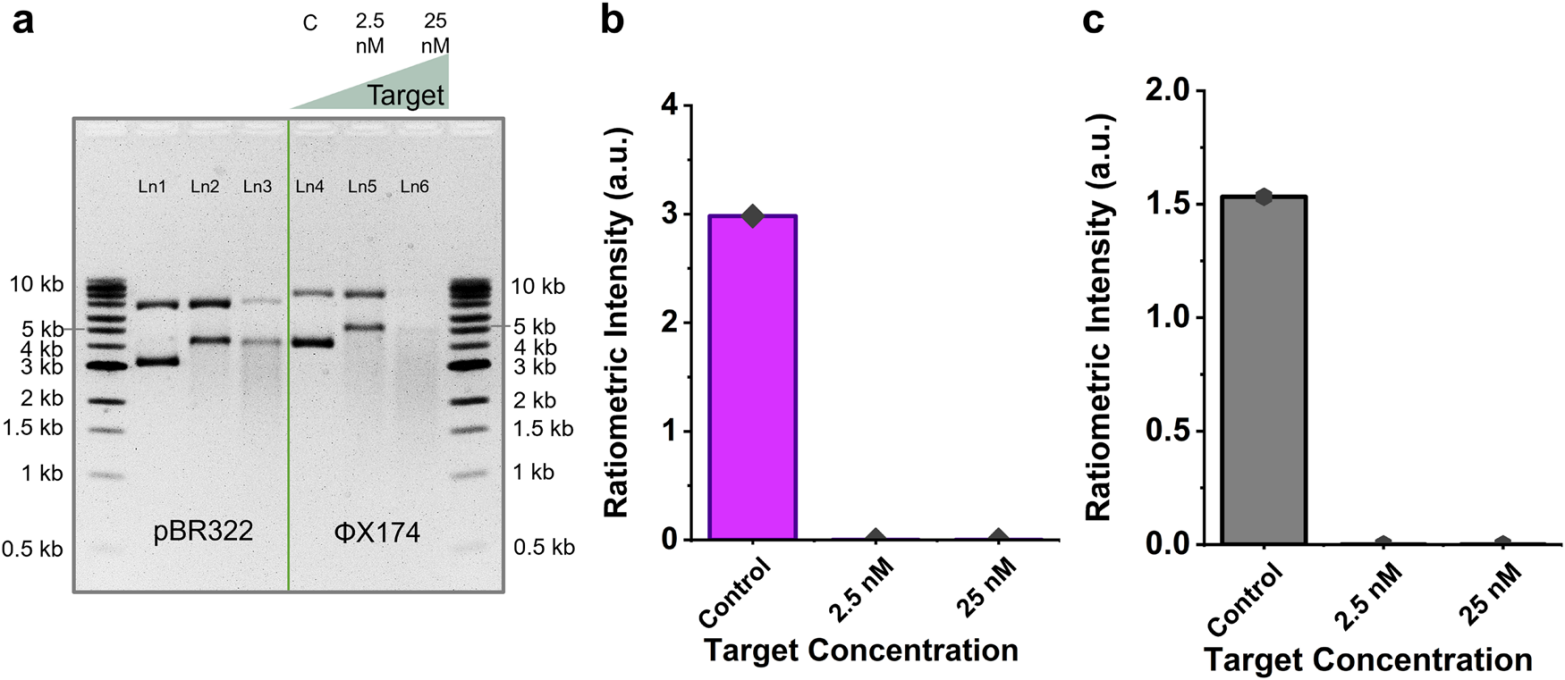
Ratiometric signal from various dsDNA plasmids. **a** Gel electrophoresis (1% agarose gel and 1× TBE buffer) results demonstrating CRISPR-Cas12a assay using dsDNA pBR322 and ΦX174 plasmids as nonspecific substrates. Bar charts demonstrating the ratiometric CRISPR signal (ratio of supercoil to circular DNA) for (**b**) pBR322, and (**c**) ΦX174 plasmid DNA. Intensity plots of this gel in (**a**) have been shown in Supplementary Fig. 3. Abbreviations: nM nanomolar, kb kilobase pair, Ln lane, TBE Tris-borate EDTA, dsDNA double-stranded DNA, a.u. arbitrary unit.

While the *trans*-nicking of pUC19 DNA is interesting, we performed additional CRISPR reactions using a few other ds plasmids, such as pBR322 and ΦX174, to see if this supercoil relaxation mechanism is widely applicable among different plasmid DNA. Figure 3a shows that the supercoil band (lower band) of pBR322 completely disappeared in the presence of 2.5 nM and 25 nM target concentrations (left panel, lanes 2 and 3). The same phenomenon was observed for ΦX174 (right panel, lanes 5 and 6). Since supercoiled bands of both plasmids disappeared with 2.5 nM and 25 nM target, the quantitative ratiometric intensity (ratio of supercoil to relaxed circular) was zero for all concentrations except for the controls (Fig. 3b, c). These results suggest that the ratiometric sensing strategy could be employed using various ds plasmid DNAs, which makes this method universal. However, the detection sensitivity would depend on the specific plasmid reporter, which is discussed in the later section.

### Assay optimization, characterization, and comparison with other reporting strategies

Among the three dsDNA plasmid reporters (pUC19, pBR322, and ΦX174), we chose pUC19 to optimize the assay conditions, and then calculated the LOD of the nonfluorescent rCRISPR assay using all three plasmid DNAs. Finally, we compared the performance of rCRISPR with the conventional fluorescent CRISPR assay based on a F-Q reporter.

**Fig. 4:**
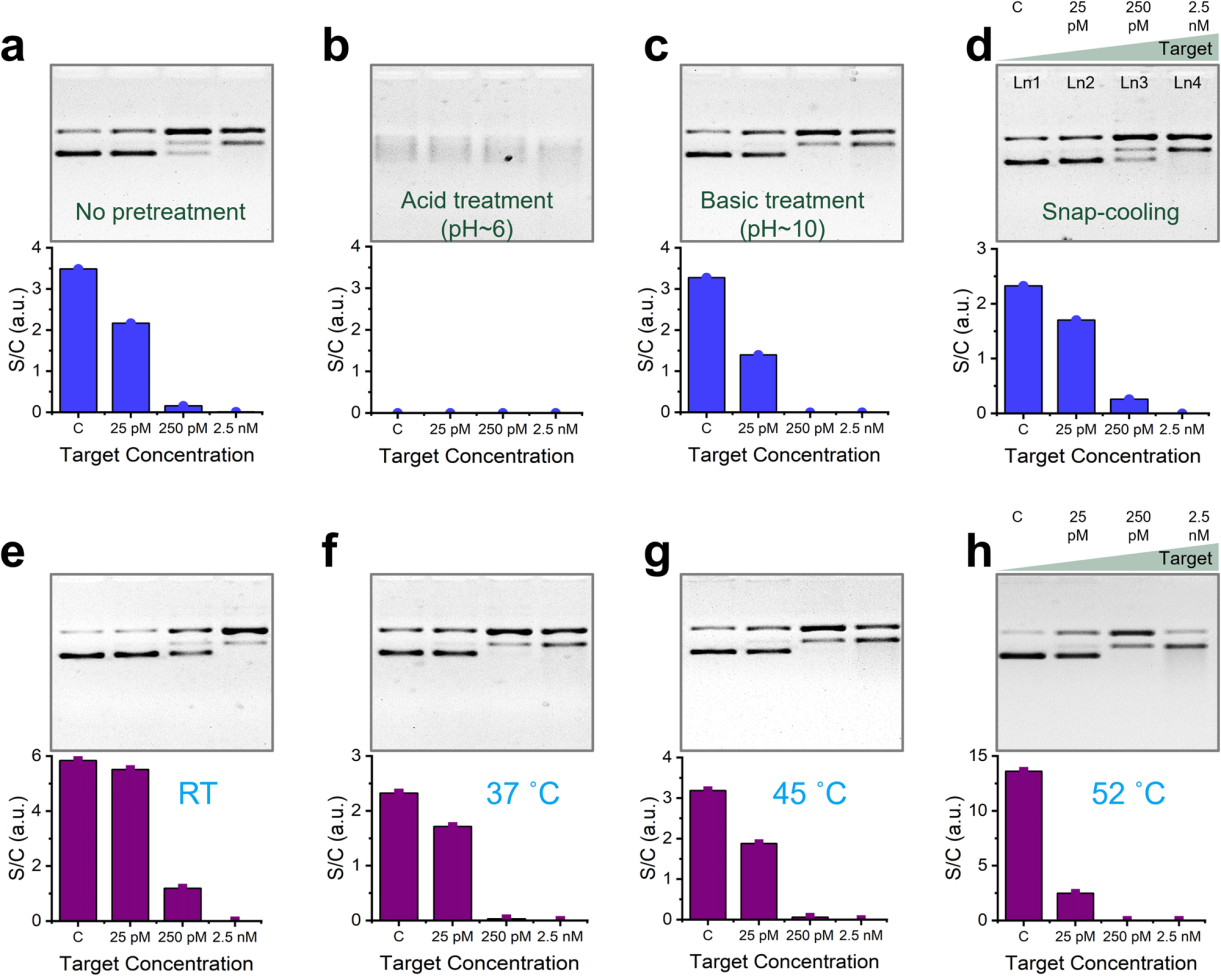
Effect of reporter pretreatment and reaction temperature. Gel electrophoresis (1% agarose gel and 1× TBE buffer) results and bar charts demonstrating the effect of no pretreatment (**a**), acid pretreatment (**b**), basic pretreatment (**c**), and snap-cooling (**d**) on *trans*-nicking of pUC19. Gel electrophoresis (1% agarose gel and 1× TBE buffer) results (top) and bar charts (bottom) demonstrating the effect of reaction temperature on *trans*-nicking of pUC19 (**e**–**h**). Intensity plots of each gel have been shown in Supplementary Fig. 4. Abbreviations: c negative control, pM picomolar, nM nanomolar, R.T. room temperature, TBE Tris-borate EDTA, a.u. arbitrary unit, S/C supercoil/relaxed circular.

We started our optimization procedure with the pretreatment of the reporter molecules. We ran rCRISPR reactions with untreated pUC19, acidic pUC19 (pH ~6), basic pUC19 (pH ~10), and snap-cooled^17^ pUC19. The gel images and bar charts in Fig. 4a–d reveal that there was no effect of snap cooling on the DNA supercoil relaxation efficiency; however, acidic condition destabilized the plasmid reporter (Fig. 4b), and basic pretreatment promoted *trans*-nicking and supercoil relaxation (Fig. 4c). Plasmid DNA is unstable under acidic conditions because low pH promotes the nucleophilic attack of the phosphate group by water molecules, resulting in the cleavage of the phosphodiester bonds^69^, causing strand breakage and thus degrading the DNA molecule. For basic treatment, we observed that the supercoil band completely disappeared with 250 pM target concentration (Fig. 4c, lane 3). At higher pH, the phosphate groups on the DNA backbone become deprotonated, increasing the DNA molecule’s negative charge density. This increased negative charge can repel the negatively charged phosphate groups, destabilizing the DNA helix and unwinding the ds structure^70^. This unwound ds portion of supercoil DNA could create easy access for the *trans*-active Cas12a protein.

Figure 4e–h show the effect of reaction temperature on DNA supercoil relaxation efficiency. We observed that 250 pM target resulted in the complete disappearance of supercoil bands for all reaction temperatures except for R.T. (Fig. 4e–h, top panels, lane 3). We concluded that R.T. is the least active reaction temperature among the temperatures we tested. In addition, we observed that higher reaction temperature generally favors the DNA supercoil relaxation (see results of 25 pM target in Fig. 4e–h, bottom panels). However, we cannot increase the reaction temperature beyond Cas12a’s stability temperature, which is about 50 °C. After that, Cas12a will be denatured and lose its biological function^71^.

**Fig. 5:**
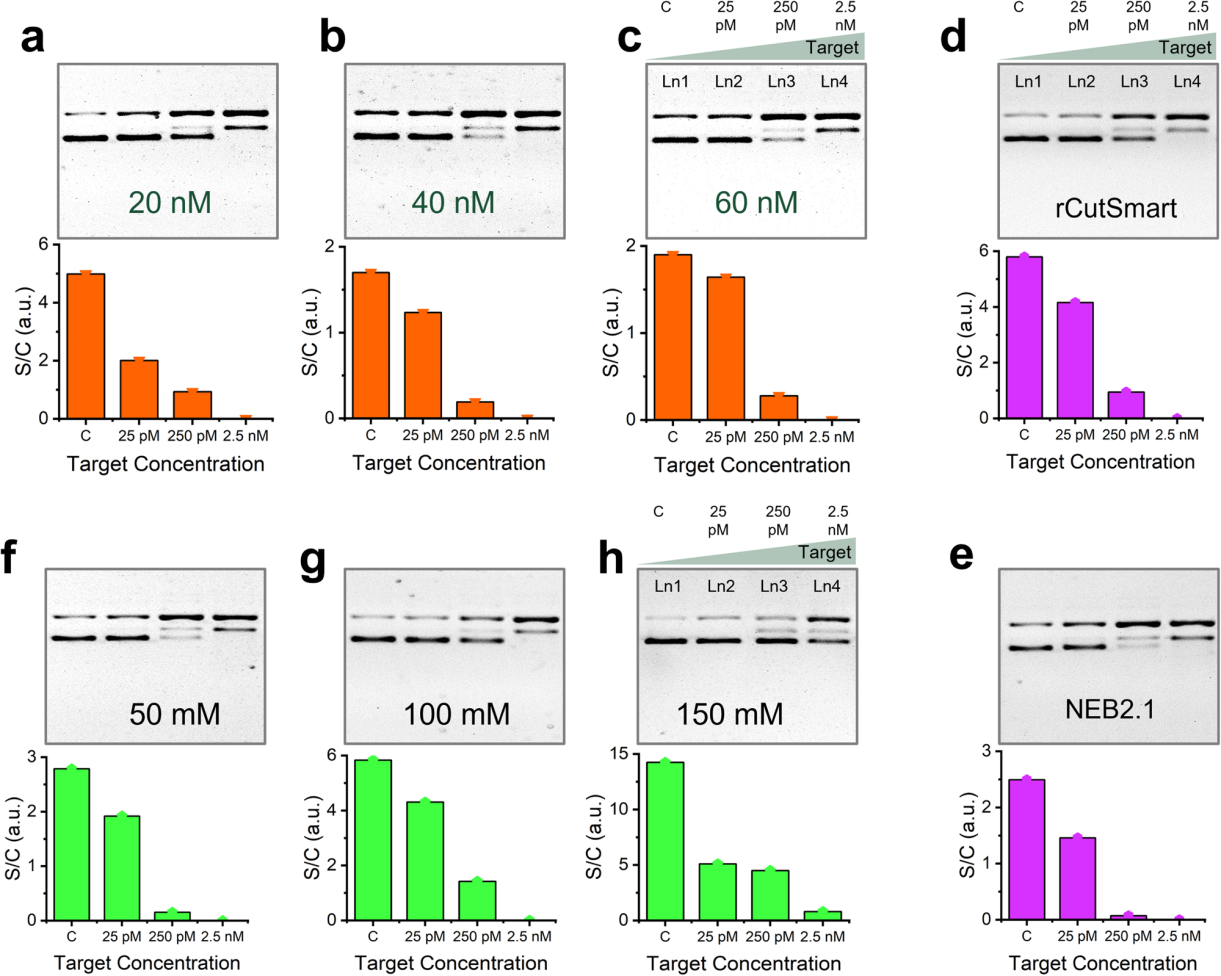
Effect of Cas12a concentration, buffer type, and salt concentration. Gel electrophoresis (1% agarose gel and 1× TBE buffer) results and bar charts demonstrating the effect of 20 nM Cas12a (**a**), 40 nM Cas12a (**b**), and 60 nM Cas12a (**c**) concentration; effect of rCutSmart reaction buffer (**d**), and NEB2.1 reaction buffer (**e**); effect of 50 mM NaCl (**f**), 100 mM NaCl (**g**), and 150 mM NaCl (**h**) on *trans*-nicking efficiency of Cas12a towards pUC19. Intensity plots of each gel have been shown in Supplementary Fig. 5. [Cas12a] = 40 nM for **d**–**h**. rCutSmart buffer recipe: 50 mM Potassium Acetate, 20 mM Tris-acetate, 10 mM Magnesium Acetate, 100 µg per ml Recombinant Albumin, pH 7.9@25 °C. NEBuffer 2.1 buffer recipe: 50 mM NaCl, 10 mM Tris-HCl, 10 mM MgCl_2_, 1 mM DTT, 100 µg per ml BSA, pH 7.9@25 °C. Abbreviations: c negative control, pM picomolar, nM nanomolar, mM millimolar, TBE Tris-borate EDTA, a.u. arbitrary unit, BSA bovine serum albumin.

Next, a series of reaction mixture conditions were optimized, including the concentration of ribonucleoprotein complex (RNP) and buffer composition. Figure 5a–c show the gel results (top) and quantitative bar charts (bottom) of three sets of rCRISPR reactions done with different Cas12a concentrations (e.g., 20 nM, 40 nM, and 60 nM). For each Cas12a concentration, we kept the same molar ratio of Cas12a:gRNA (2:1) according to the literature^45^. For each set, 25 pM, 250 pM, and 2.5 nM target concentrations, including a negative control, were tested. We observed that the supercoiled band (Fig. 5a–c, top panels, lane 3) for 250-pM target became thinner with the increase of Cas12a concentration, which indicates that higher Cas12a concentration performed better for DNA supercoil relaxation. However, if we compare the intensity differences between negative control and 25-pM target of each set (Fig. 5a–c, bottom panels), we observed that the maximum bar height difference was obtained for 20 nM Cas12a concentration. The results suggest that the optimal Cas12a concentration depends on the concentration of the target nucleic acid. In general, lower RNP concentrations are more suitable for detecting lower concentrations of target nucleic acids, while higher Cas12a concentrations are preferable for detecting higher target concentrations, as we previously observed^15^. However, for real samples since we would not know the actual target concentrations, we chose intermediate Cas12a concentration (40 nM) for all later experiments unless otherwise mentioned.

Besides, we explored the effect of reaction buffer types and salt concentration (ionic strength) on DNA supercoil relaxation. We used rCutSmart buffer and NEBuffer2.1, containing two different types of salts: acetate salt vs. chloride salt (buffer compositions are mentioned in the caption of Fig. 5). Both qualitative (Fig. 5d, e, top panels, lane 3) and quantitative (Fig. 5d, e, bar charts for 250 pM target) results suggest that NEBuffer2.1 performs better. In addition, we explored the effect of salt concentration on the *trans*-nicking of supercoil DNA (Fig. 5f–h). We observed that higher salt concentrations (e.g., 150 mM NaCl) resulted in a lower nicking rate (Fig. 5f–h, top panels, lanes 3 and 4). A higher concentration of cations such as Na^+^ is known to stabilize nucleic acid helix at hybridized state^72^. Thus, plasmids became more immune from being nicked at higher ionic strength.

**Fig. 6:**
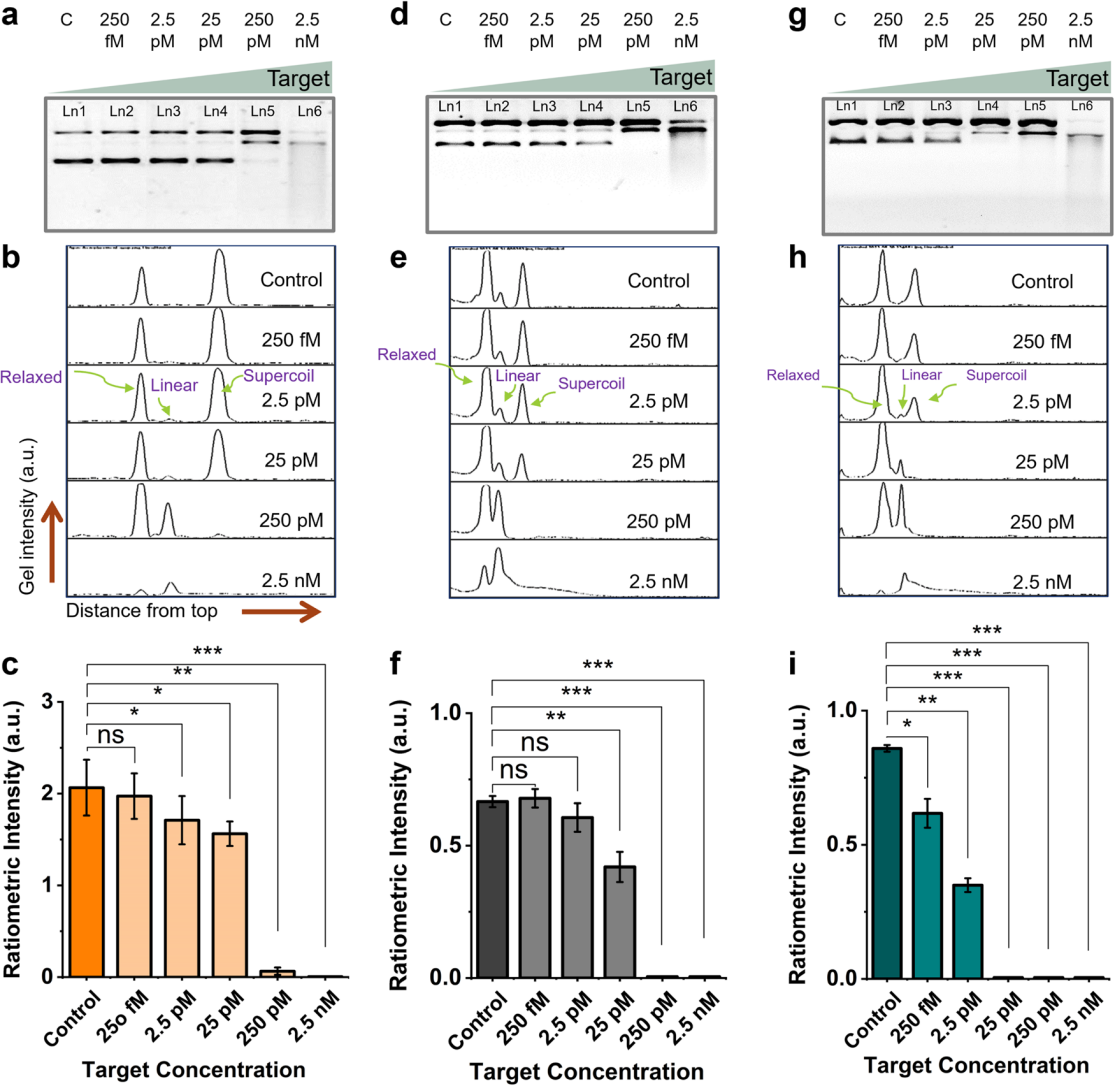
Limit of detection (LOD) determination. Gel electrophoresis (1% agarose gel and 1× TBE buffer) demonstrating the CRISPR-Cas12a induced DNA relaxation for various target concentrations using pUC19 (**a**), pBR322 (**d**), and ΦX174 (**g**) reporters, respectively. Gel intensity diagrams of gel lanes for pUC19 (**b**), pBR322 (**e**), and ΦX174 (**h**) reporters. Ratiometric intensity plotted in bar chart against different target concentrations for pUC19 (**c**), pBR322 (**f**), and ΦX174 (**i**) reporters. These assays were performed at 52 °C for 1 h. Error bar represents the standard deviation of *n* = 3 repeated experiments for each measurement. The graph shows statistical insignificance at *p* > 0.05 (ns), significance at *p* < 0.05 (*), *p* < 0.01 (**), and *p* < 0.001(***). Abbreviations: c negative control, fM femtomolar, pM picomolar, nM nanomolar, Ln lane, TBE Tris-borate EDTA, a.u. arbitrary unit.

After the assay optimization, we characterized the LOD of the rCRISPR assay at optimized conditions (e.g., basic pretreatment, T = 52 °C, NEB2.1 buffer, etc.). We first characterized the LOD for pUC19 reporter. We titrated the assay with a broad range of analyte concentrations, ranging from 250 fM to 2.5 nM for 1 hr of reaction. The gel image is shown in Fig. 6a. We observed that the supercoil band of pUC19 reporter gradually became thinner, and the relaxed circular band became stronger. Besides, a new band for linear reporter molecules started to appear with the increasing target concentrations (Fig. 6a, lanes 5 and 6). We plotted intensity diagrams for each lane of the gel (Fig. 6b). The left peak denotes the relaxed circular form, the middle one is linear DNA, and the right peak is for supercoiled pUC19 DNA. We measured the peak intensity and plotted it in a bar chart against various target concentrations (Fig. 6c). We performed statistical analysis (t-test) to determine the minimum concentration we could detect with our newly proposed ratiometric sensing strategy. We estimated a LOD of ~2.5 pM for the synthetic ssDNA target based on statistical analysis.

Furthermore, we repeated the experiments using pBR322 (Fig. 6d–f) and ΦX174 (Fig. 6g–i) as the reporter molecules for varied target concentrations from 250 fM to 2.5 nM. We found LODs of 25 pM and 250 fM for pBR322 and ΦX174 reporter, respectively, using a similar analysis as mentioned above. To bolster our result, we chose another synthetic ssDNA target (name: Target ssDNA_1, see the sequence in Supplementary Table 1) and performed rCRISPR assay. This time, we achieved even better detection results (Supplementary Figs. 6 and 7), which is not surprising because the sensitivity could depend on the sequence of target (or gRNA)^64,73^. We achieved LODs of 200 fM for pUC19 and pBR322, and 100 fM for ΦX174 reporter when we used target ssDNA_1 (Supplementary Fig. 7). We observed that ΦX174 outperformed the other two plasmid reporters we tested in detecting different targets. In this ratiometric sensing strategy, the sensitivity primarily depends on how easily a supercoiled DNA gets converted to the relaxed state. Therefore, if a reporter plasmid has a higher initial supercoiled fraction or a certain supercoiled plasmid requires fewer number of nicking, that plasmid would likely give a more sensitive detection result. Previous studies show that different circular plasmids have varying linking number/degree of supercoiling and thermodynamic stability at supercoiled state^74–77^. The ease of supercoil relaxation might also be influenced by the structural and compositional differences (such as G.C. content, repeat regions etc.) among the plasmids, which required further investigation. At this point, it is unclear whether sequence difference or disparities in supercoiling makes CRISPR-Cas12a-induced supercoil relaxation favorable. However, we suspect that ΦX174, compared to pUC19 and pBR322, might require fewer number of nicking to convert it into relaxed form. It is also very likely that an even better-performing plasmid DNA than ΦX174 may exist. The search for the best ds plasmid reporter is however beyond the scope of this study. Nevertheless, there are a few potential strategies to make this ratiometric sensing strategy more efficient in future. For instance, using DNA topoisomerases (e.g., Topo I, DNA gyrase, Topo III, and Topo IV), we can change the linking number of plasmids to get the best supercoil state that would require the least number of cutting to convert it into relaxed conformation^75^. Also, DNA gyrase enzyme could be utilized to increase the amount of supercoil DNA in the initial sample^78^, which will help set up a high starting ratiometric value for the best contrast.

Next, we compared our results with a typical fluorescent reporting system and found that our ratiometric reporting strategy performed two orders of magnitudes better than the widely used F-Q reporters (Supplementary Fig. 8). Besides, this ratiometric sensing strategy is advantageous from a few experimental perspectives while comparing it to our previously demonstrated sizing-based detection: 1) Our previous λ DNA-based reporting system requires a reference lane of unreacted λ DNA to quantify band shift. Still, measurement errors could arise because of gel distortion, misalignment, uneven heat distribution and uneven distribution of electric fields across gel width and running buffer level. Instead, in our current plasmid-based reporting system, we measure ratiometric band intensity instead of band position, which is independent of gel distortion, misalignment, and running buffer level. 2) In most of the assay methods, including our previous sizing-based signaling mechanism, errors may also arise from the pipetting when adding reporter molecules. Our current strategy is completely immune from this type of human error, because the intensity ratio of supercoiled to relaxed DNA is independent of the absolute amount of reporter molecules added to each reaction. For a thorough comparison, we have included a comprehensive summary table (Supplementary Table 2), juxtaposing our current reporting strategy with established various F-Q ssDNA reporters (F-TTATT-Q, F-TTATT-5C-Q, F-8C-Q, F-10C-Q)^10,79,80^, SPR ssDNA^60,61^, F-Q dsDNA reporters, and various DNA sizing-based reporters. While SPR ssDNA reporting provided the best sensitivity because of the nature of SPR, we found that the DNA supercoil relaxation technique excels most of previous reporting systems in terms of cost, speed, and sensitivity. Furthermore, DNA supercoil relaxation can easily be evaluated using a more miniaturized microfluidic electrophoresis system^81,82^ instead of standard gel electrophoresis, showing the potential of this method for future POC use like our previous sizing-based methods.

### Validation of ratiometric sensing strategy using real-life targets

**Fig. 7:**
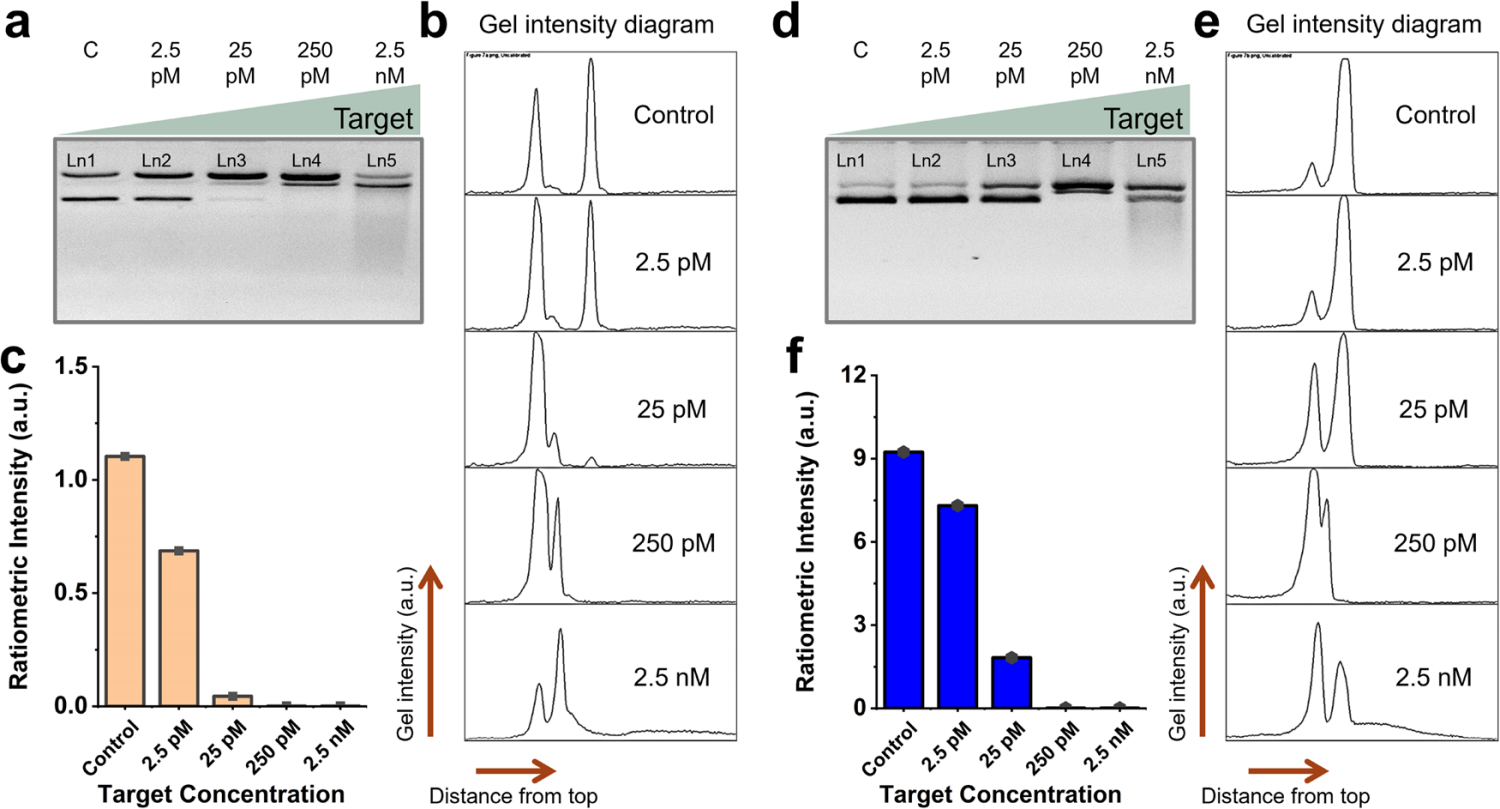
Validation of CRISPR-Cas12a based DNA supercoil relaxation using various targets. Gel electrophoresis (1% agarose gel and 1× TBE buffer) demonstrates the nonspecific supercoil relaxation of ΦX174 DNA; **a** Marker of AAV genome was used as target. **d** Marker of HPV 16 was used as target. In each panel in **a** and **d**, Ln1: negative control (no target); Ln2, Ln3, Ln4, and Ln5: experimental lanes with target concentrations of 2.5 pM, 25 pM, 250 pM, and 2.5 nM, respectively. These assays were performed at 52 °C for 1 h. Gel intensity diagrams of each lane for AAV (**b**), and HPV 16 (**e**) synthetic targets. Ratiometric intensity plotted in bar chart against different concentrations of AAV (**c**), and HPV16 (**f**) synthetic targets. Abbreviations: c negative control, pM picomolar, nM nanomolar, Ln lane, TBE Tris-borate EDTA, a.u. arbitrary unit.

Last but not least, we used ΦX174 as the reporter molecule and checked if the supercoil relaxation reporting mechanism could be applied to other DNA targets. First, we checked if the rCRISPR assay is also valid for dsDNA targets. For that, we used a 89-bp long dsDNA as the target (see the sequences in Supplementary Table 1) and ran the rCRISPR assay. The qualitative nicking result (in gel image), and quantitative ratiometric signals in Supplementary Fig. 9 confirmed that supercoil relaxation of ΦX174 is equally valid for dsDNA targets. Next, we chose two synthetic sequences related to viral genomes AAV^83^ and HPV 16^45^ (see the sequences in Supplementary Table 1) as the model biomarkers to test the rCRISPR assay. The gel image and quantitative analysis of it in Fig. 7 show that DNA supercoil relaxation mechanism is universally effective for both targets.

**Fig. 8:**
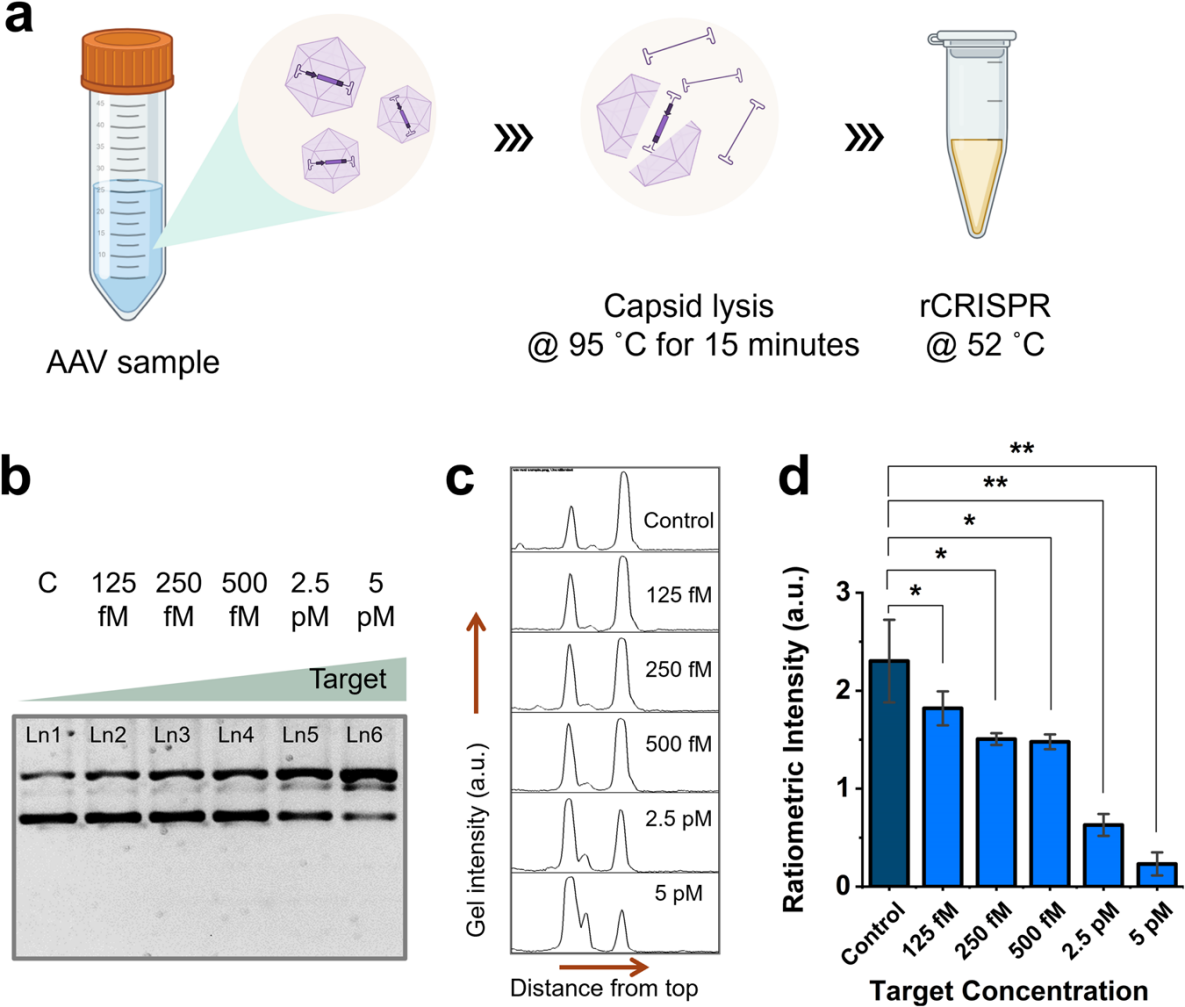
Performance of rCRISPR for detecting real AAV samples. **a** Schematic showing the procedures followed for detecting affinity-purified AAV2 samples. **b** Gel electrophoresis (1% agarose gel and 1× TBE buffer) results demonstrating the supercoil relaxation of ΦX174 DNA reporter after 1 h of rCRISPR assay using lysed AAV sample. **c** Gel intensity diagrams of each lane in **b**. **d** Quantitative performance of rCRISPR with lysed AAV sample. Error bar represents the standard deviation of *n* = 3 repeated experiments for each measurement. The graph shows statistical significance at *p* < 0.05 (*), and *p* < 0.01 (**). Abbreviations: c negative control, fM femtomolar, pM picomolar, Ln lane, TBE Tris-borate EDTA, a.u. arbitrary unit.

Finally, we evaluated the efficacy of our rCRISPR assay with real Adeno-associated virus (AAV) samples (Fig. 8). We released the viral DNA using a heat inactivation technique and then the lysed samples were detected using rCRISPR (Fig. 8a). The rCRISPR assay worked robustly with crude AAV lysates without any additional purification steps. We evaluated the LOD with real AAV samples, which was found to be ~125 fM (Fig. 8b–d). These results suggest that our simple, ratiometric sensing platform has the potential to detect various genes and pathogens.

rCRISPR is a versatile strategy that can be applied to detect a wide range of targets (e.g., ssDNA and dsDNA targets) sensitively. This method demonstrated fM detection sensitivity without preamplification steps, high reaction rate that could be completed within 10 min, and only required low-cost, label-free reporting molecules (Supplementary Table 2). In addition, this method can provide both qualitative and quantitative results. For instance, the gel results could be detected by naked eye for quick yes or no assessment (Supplementary Fig. 6g, naked eye LOD ~ 250 fM), or via a professional gel scanner for quantitative analysis (Supplementary Fig. 7i, quantitative LOD ~ 100 fM). Impressively, both approaches can achieve fM detection sensitivity, satisfying the needs for broad sensing applications.

While the initial results of rCRISPR are promising, there are a few areas that could be further improved in future in using plasmid reporters. Firstly, currently we analyzed gel results using ImageJ software manually, which may vary the quantification results from user to user. This issue could be addressed by using professional and automated gel analysis software such as PyElph^84^, GELect^85^, and EgyGene GelAnalyzer4^86^ to reduce user bias. Secondly, the assay sensitivity depends on the initial amount of supercoiled plasmid in the reporter system (Fig. 6). As such, searching for a higher initial supercoil percentage plasmid reporter or methods to increase the initial supercoil percentage could be an area of future research to further improve the detection sensitivity. Thirdly, the assay is based on the nicking and unknotting of supercoiled DNA. Therefore, we suspect that DNA ligase protein may be a potential interferent. DNA ligase might revert the plasmid relaxation by repairing the nicks and interfere with the assay signal. This inference together with others will be studied further in future experiments. Finally, for future POC diagnostic applications, the lab-based gel electrophoresis technology can be replaced with battery-powered microfluidic gel electrophoresis plus smartphone-based imaging^87^ to eliminate the need for bulky equipment.

The main finding of this research is that Cas12-induced *trans*-nicking of plasmid DNA could be used to generate sensitive (fM), rapid (<10 min), and self-calibrated (ratiometric) CRISPR assay signal that does not require expensive fluorescent labels and readout equipment. While we mainly used gel electrophoresis for measuring the percentage of supercoil and relaxed circular DNA plasmid in this study, it is possible to integrate another quantification technology (other than the gel) to measure the relative populations of supercoil and circular DNA that would ultimately lead to develop new-generation rCRISPR for potentially higher detection sensitivity and throughput.

In summary, we demonstrated rCRISPR, an interesting strategy for CRISPR-Cas12a-based biosensing by using supercoiled plasmid DNA as ratiometric reporter molecules. We discovered that even though the *trans*-degradation of ds plasmid DNA with activated Cas12a is very slow (>90 min and requires high target concentrations), the *trans*-nicking of supercoil DNA and converting it to relaxed circular conformation is a much faster process (<10 min). This interesting Cas12a-induced *trans*-nicking property has been leveraged to construct a nonfluorescent, molecular conformation-based CRISPR detection system free of expensive reporter molecules and optical readout equipment. We tested a few dsDNA plasmids, such as pUC19, pBR322, and ΦX174, and found that the DNA supercoil relaxation mechanism is widely prevalent, which makes our sensing strategy universal. Among these three plasmids, ΦX174 performed the best, which was able to detect ssDNA target with a LOD of ~100 fM without any preamplification steps, two orders better than a typical fluorescent reporting system with the same target and reagent combination. Additionally, this ratiometric signaling mechanism obviates all limitations of the previously demonstrated sizing-based signal acquisition technique without compromising sensitivity. As a proof-of-concept, we also applied DNA supercoil relaxation to detect AAV and HPV 16 as model targets. This straightforward yet sensitive strategy is compatible with low-cost and compact readout systems such as microfluidic/mini-gel electrophoresis, making it appealing for POC implementation in the future.

## Methods

### Chemicals and apparatus

LbaCas12a, crRNA, synthetic target, and crRNA were purchased from Integrated DNA Technologies (IDT, Coralville, IA, USA). rCutSmart™ buffer, and NEBuffer™ 2.1, pUC19 DNA (Catalog# FERSD0061), pBR322 DNA (Catalog# FERSD0041 and phiX174 DNA (Catalog# FERSD0031), were purchased from Thermo-fisher. Ultrapure water (18.3 MΩ cm) was produced by the Milli-Q system (Millipore, Inc., USA) and used throughout the experiments. The sequences of oligonucleotides and crRNA molecules are listed in Supplementary Table 1.

### Nonfluorescent LbaCas12a/gRNA assays

All DNA and RNA oligos were stored in 10 mM Tris-HCl buffer (pH 7.5) at 10 μM concentrations in a −20 °C refrigerator and pre-warmed 37 °C for 30 min before mixing. Reporter molecules (e.g., 1 kb dsDNA, pUC19 DNA, pBR322, phiX174) were diluted to 100 ng per µL in 10 mM Tris-HCl buffer (pH 7.5), stored at 4 °C and used throughout the experiments. For the nonfluorescent CRISPR assay, analyte solutions with different concentrations of ssDNA were added into the CRISPR reaction reagents, consisting of 40 nM LbaCas12a, 20 nM gRNA, 4 ng per µL reporter molecule and 1X reaction buffer (e.g., 50 mM NaCl, 10 mM Tris-HCl, 10 mM MgCl_2_, 100 µg per ml BSA, pH = 7.9 @ 25 °C). The total reaction volume was 40 µL. The assay was performed at 37 °C for 1 h. This protocol was followed for all experiments unless otherwise mentioned.

### Fluorescent readout

For the fluorescent readout system, 600 nM F-Q reporter molecule (see the sequence in Supplementary Table 1) was used instead of dsDNA plasmid. The CRISPR reaction mixture was incubated in a microplate reader (SpectraMax M2, Molecular Devices) at 37 °C for 1 h with fluorescence measurements (excitation at 490 nm, and emission at 520 nm) taken every 30 s.

### AAV sample preparation

The affinity-purified AAV2 (Millipore Sigma) underwent pretreatment through capsid lysis at 95 °C for 15 min. Prior to performing rCRISPR, AAV dilutions were prepared in 1× DNase buffer (Invitrogen) supplemented with 0.001% Pluronic F-68.

### Nonfluorescent readout using gel electrophoresis

Gel electrophoresis was performed for the nonfluorescent reporting system. 1% agarose gels were prepared using 1× TBE (Tris Borate EDTA) and 1× SYBR™ Safe DNA Gel Stain (Thermo-fisher, catalog# S33102). 10 μL of different reaction products with loading dye (5:1, v/v) were added to each well. Electrophoresis was done at 120 V for 30 min in the same buffer at room temperature. Finally, the agarose gels were scanned and recorded by the E-Gel Imager system (Invitrogen, USA).

### Gel result analysis

Gel results were analyzed using ImageJ software. First, the background was removed from the gel image. The longitudinal intensity of each lane was averaged across the width and plotted. Gel intensity was calculated from the distinct peaks for supercoiled and relaxed circular conformations. Ratiometric intensities (aka. Ratio of supercoiled DNA to relaxed circular DNA) were calculated by dividing supercoiled band intensity by relaxed circular band intensity. Finally, the ratiometric intensities were plotted against the target concentration.

### Statistical analysis

We performed the Student’s *t* test using repeated experimental data (*n* = 3) to calculate the *p* values to estimate the difference between the experimental results and control groups and determined the LOD. The graph shows statistical insignificance at *p* > 0.05 (ns), and statistical significance at *p* < 0.01 (*), *p* < 0.01 (**), and *p* < 0.001(***).

### Reporting summary

Further information on research design is available in the Nature Portfolio Reporting Summary linked to this article.

### Supplementary information

Supporting material

Reporting Summary

## Data Availability

Data presented in this study can be found in the article and supplementary file. Additional data are available from the corresponding author upon reasonable request.
