# Supplementary material for: Ratiometric nonfluorescent CRISPR assay utilizing Cas12a-induced plasmid supercoil relaxation
Source: Commun Chem. 2024 Jun 8;7:130. doi: 10.1038/s42004-024-01214-2 (PMC11162422; doi:10.1038/s42004-024-01214-2)
Supplement: Supplementary file 1 — Supporting material [file 42004_2024_1214_MOESM1_ESM.pdf]

Supplementary Information

Ratiometric Nonfluorescent CRISPR Assay Utilizing Cas12a-Induced Plasmid Supercoil Relaxation

Noor Mohammad,[1,2] Logan Talton,[1] Selen Dalgan,[1] Zach Hetzler,[1] Anastasiia Steksova,[1] and Qingshan Wei[1]*

1. Department of Chemical and Biomolecular Engineering, North Carolina State University, Raleigh, NC 27695, USA
2. Department of Chemical Engineering, Bangladesh University of Engineering and Technology, Dhaka 1000, Bangladesh

* Corresponding email: qwei3@ncsu.edu

Supplementary Discussion

1. Supplementary Figures

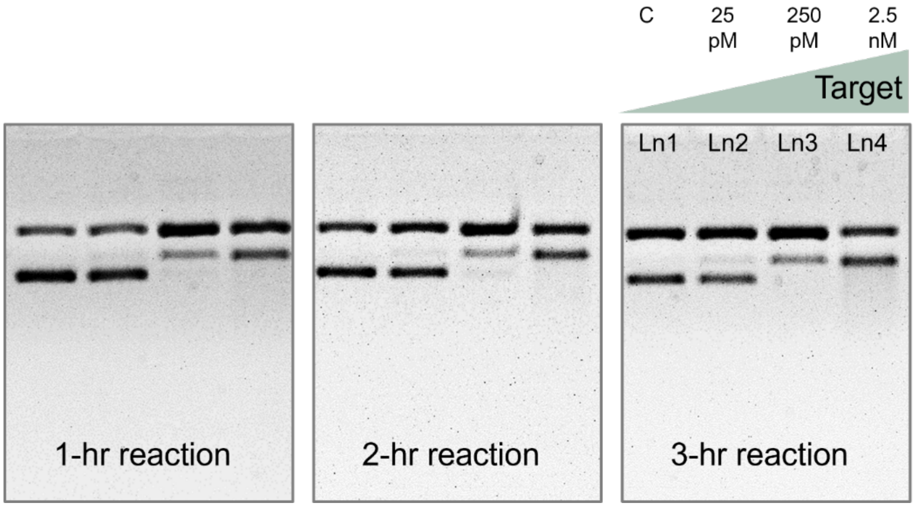

Supplementary Figure 1. Gel electrophoresis (1% agarose gel and 1×TBE buffer) results demonstrating the effect of reaction time on *trans*-nicking of pUC19 reporters. Abbreviations: c, negative control; pM, picomolar; nM, nanomolar; TBE, Tris-borate EDTA.

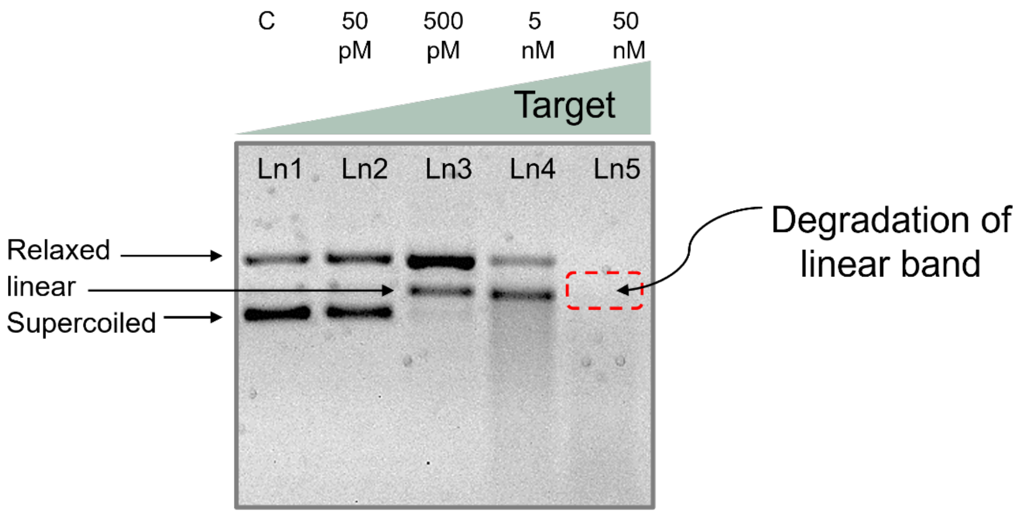

Supplementary Figure 2. Gel electrophoresis (1% agarose gel and 1×TBE buffer) results demonstrating 1-hr CRISPR-Cas12a induced *trans*-nicking of pUC19 for various target concentrations. At 50 nM target, linear DNA was degraded completely (Ln 5). Abbreviations: c, negative control; pM, picomolar; nM, nanomolar; Ln, lane; TBE, Tris-borate EDTA.

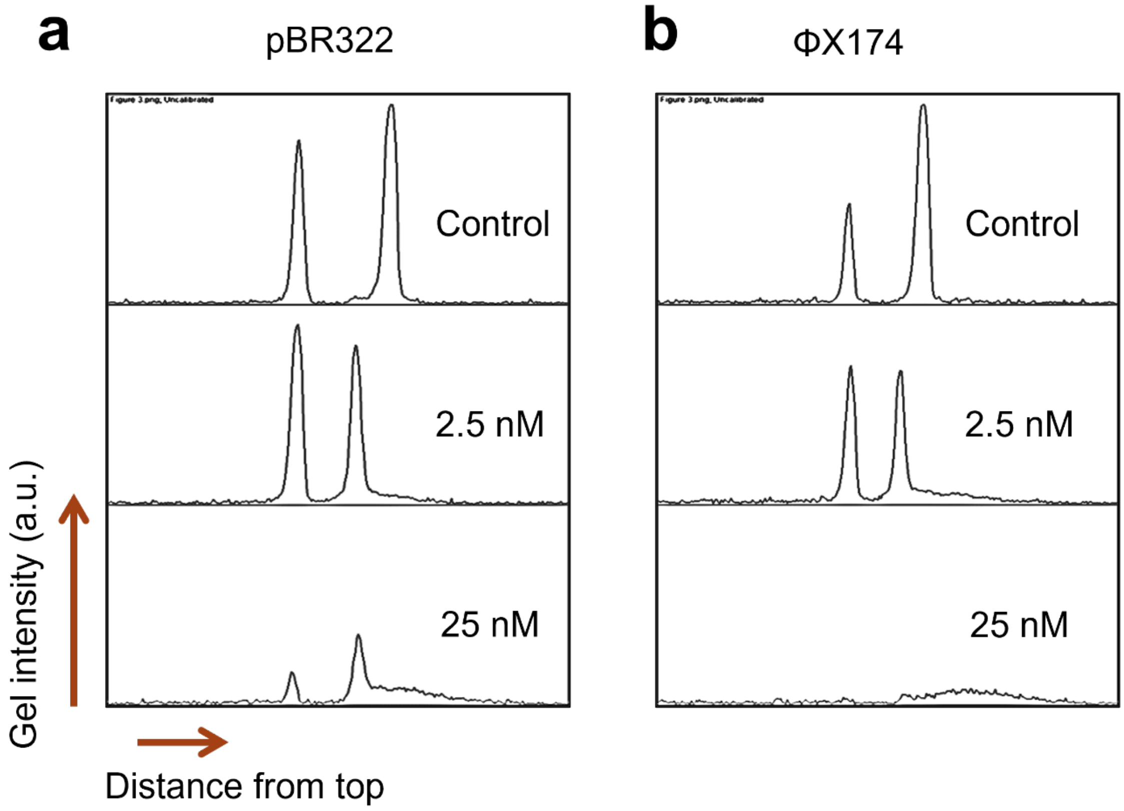

Supplementary Figure 3. Intensity diagrams for (a) pBR322, and (b) ΦX174 plasmid DNA reporter. These diagrams were generated using the gel image of **Fig**. 3a. Abbreviations: nM, nanomolar; a.u., arbitrary unit.

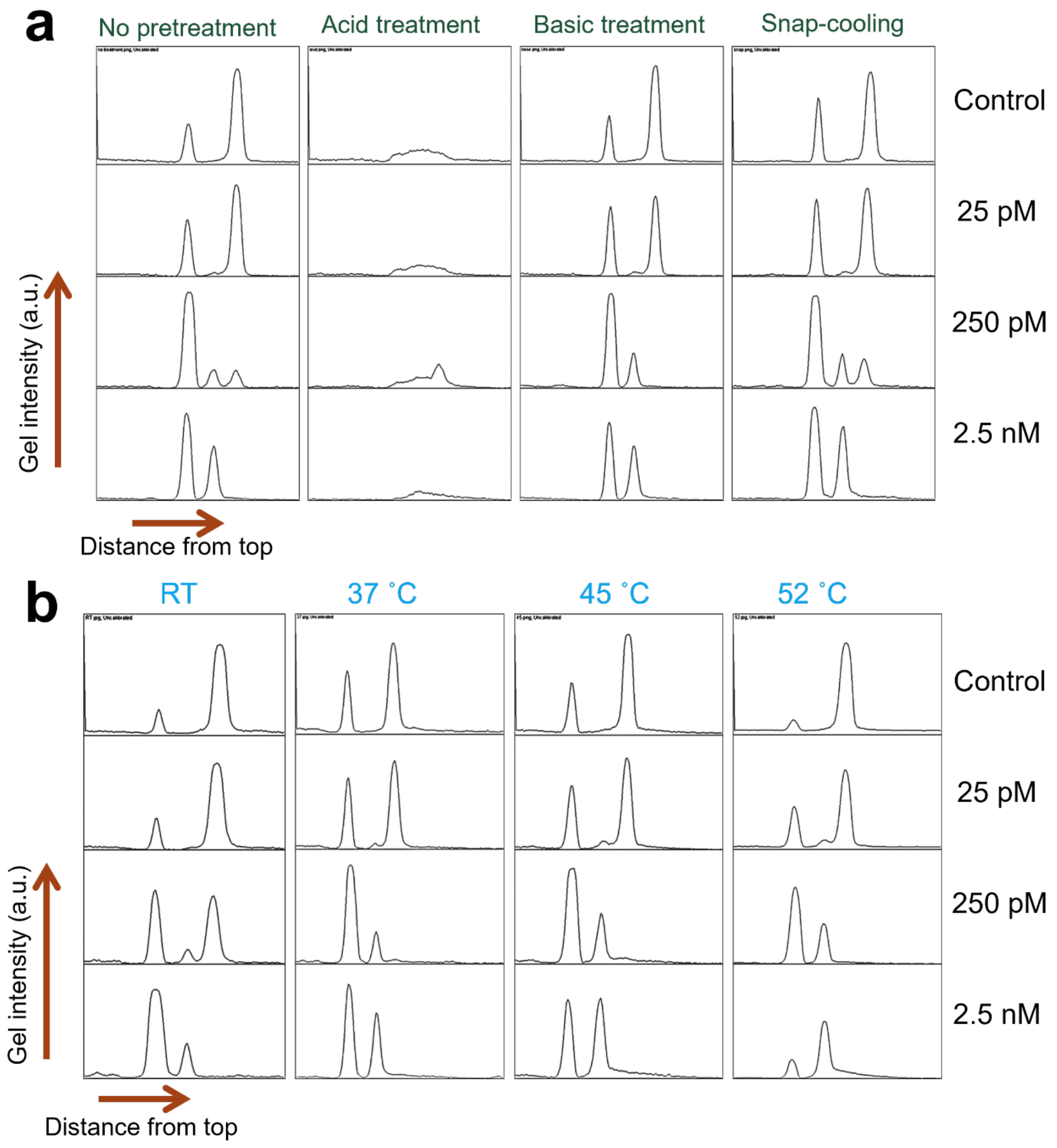

Supplementary Figure 4. Intensity diagrams of gel results to show the effect of (a) pretreatment of reporter molecule, and (b) reaction temperature. Abbreviations: c, negative control; pM, picomolar; nM, nanomolar; RT, room temperature; a.u., arbitrary unit.

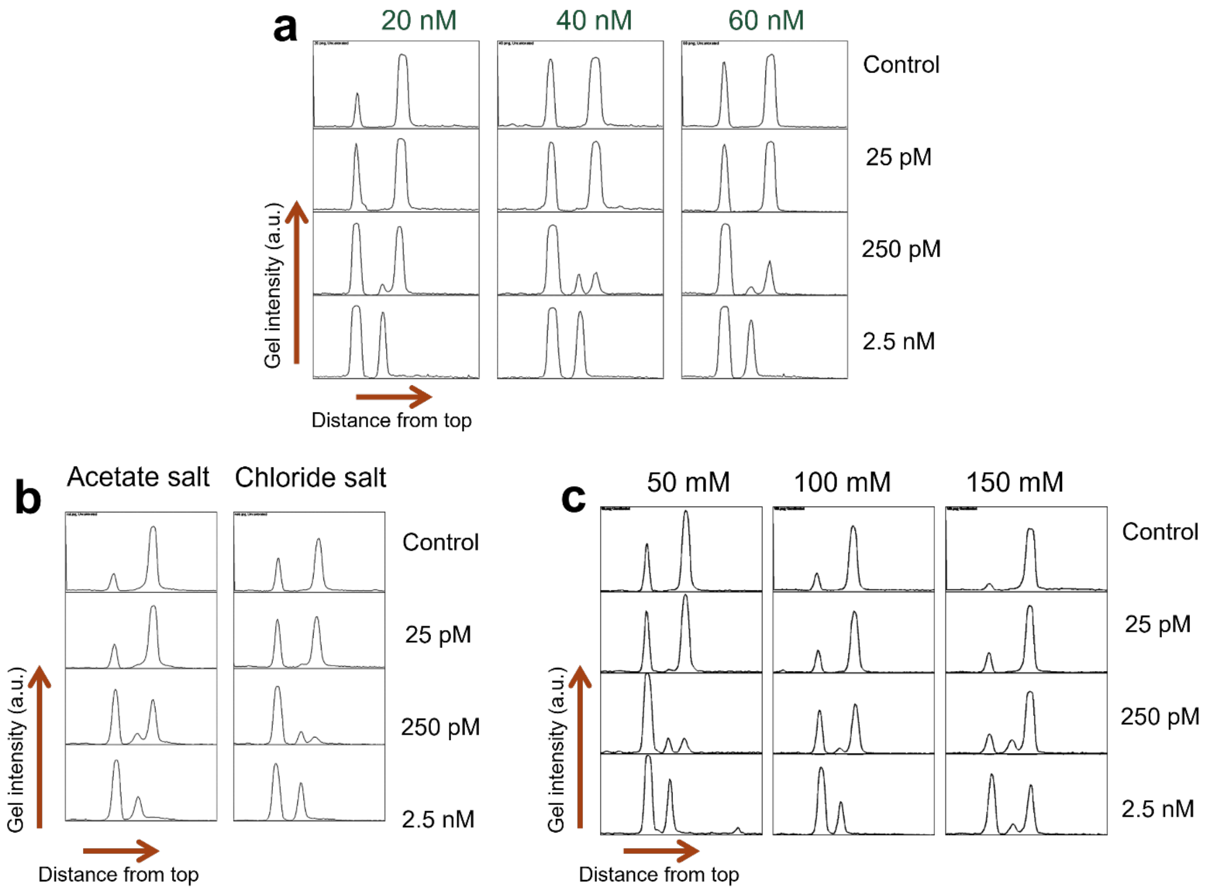

Supplementary Figure 5. Intensity diagrams of gel results to show the effect of (a) Cas12a concentration, (b) buffer type, and (c) salt concentration. Abbreviations: c, negative control; pM, picomolar; nM, nanomolar; mM, millimolar; a.u., arbitrary unit.

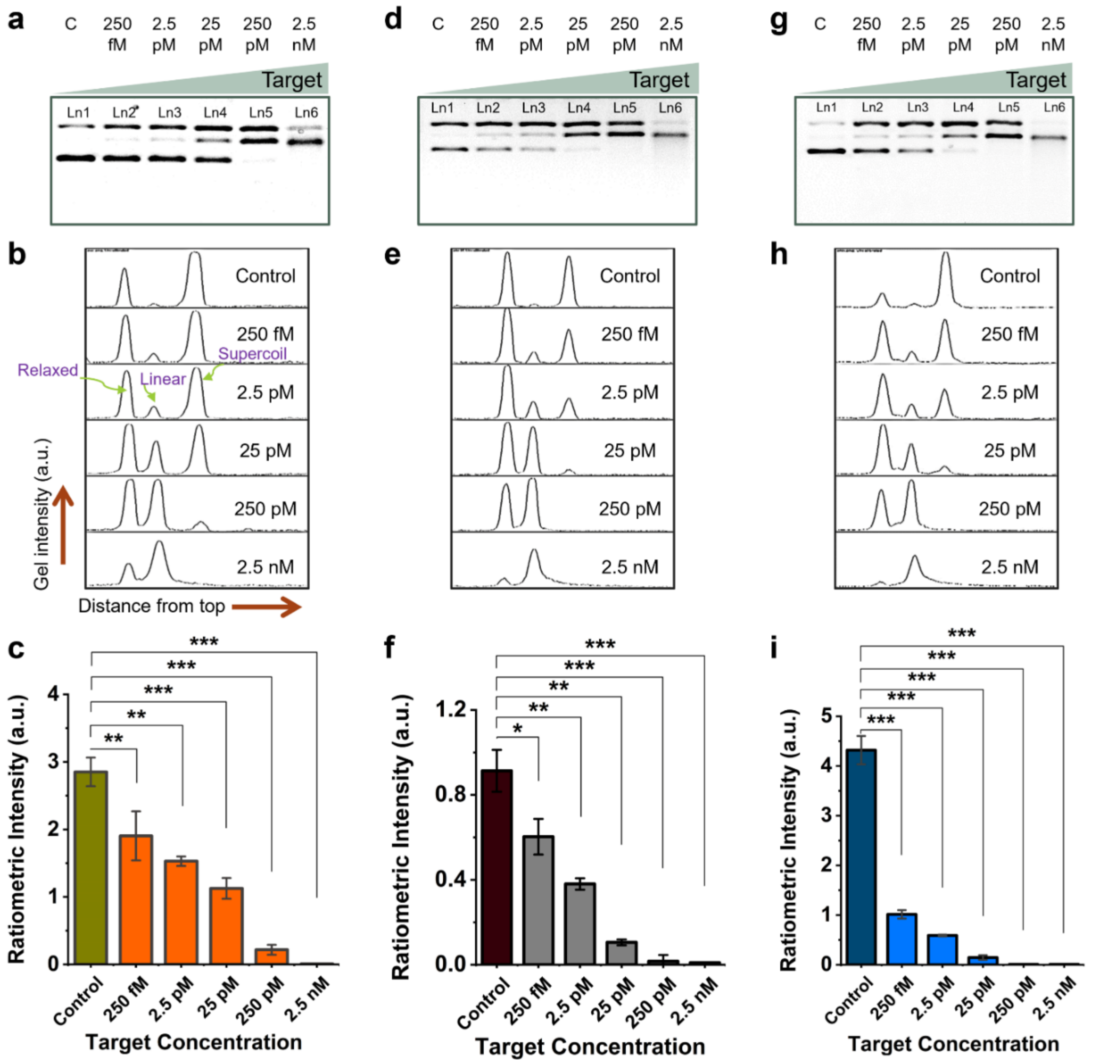

Supplementary Figure 6. Performance of various plasmid reporters with synthetic ssDNA target (Target ssDNA_1, see the sequence in Tabe S1). Gel electrophoresis (1% agarose gel and 1×TBE buffer) demonstrating the CRISPR-Cas12a induced supercoil relaxation for various target concentrations using pUC19 (a), pBR322 (d), and ΦX174 (g) reporters. Gel intensity diagrams of each lane for pUC19 (b), pBR322 (e), and ΦX174 (h) reporters. Ratiometric intensity plotted in bar chart against different target concentrations for pUC19 (c), pBR322 (f), and ΦX174 (i) reporting systems. The assays were performed at 52 ˚C for 1 hour. Error bar represents the standard deviation of n=3 repeated experiments for each measurement. The graph shows statistical significance at p<0.01 (*), p<0.01 (**), and p<0.001(***). Abbreviations: c, negative control; fM, femtomolar; pM, picomolar; nM, nanomolar; Ln, lane; TBE, Tris-borate EDTA; a.u., arbitrary unit.

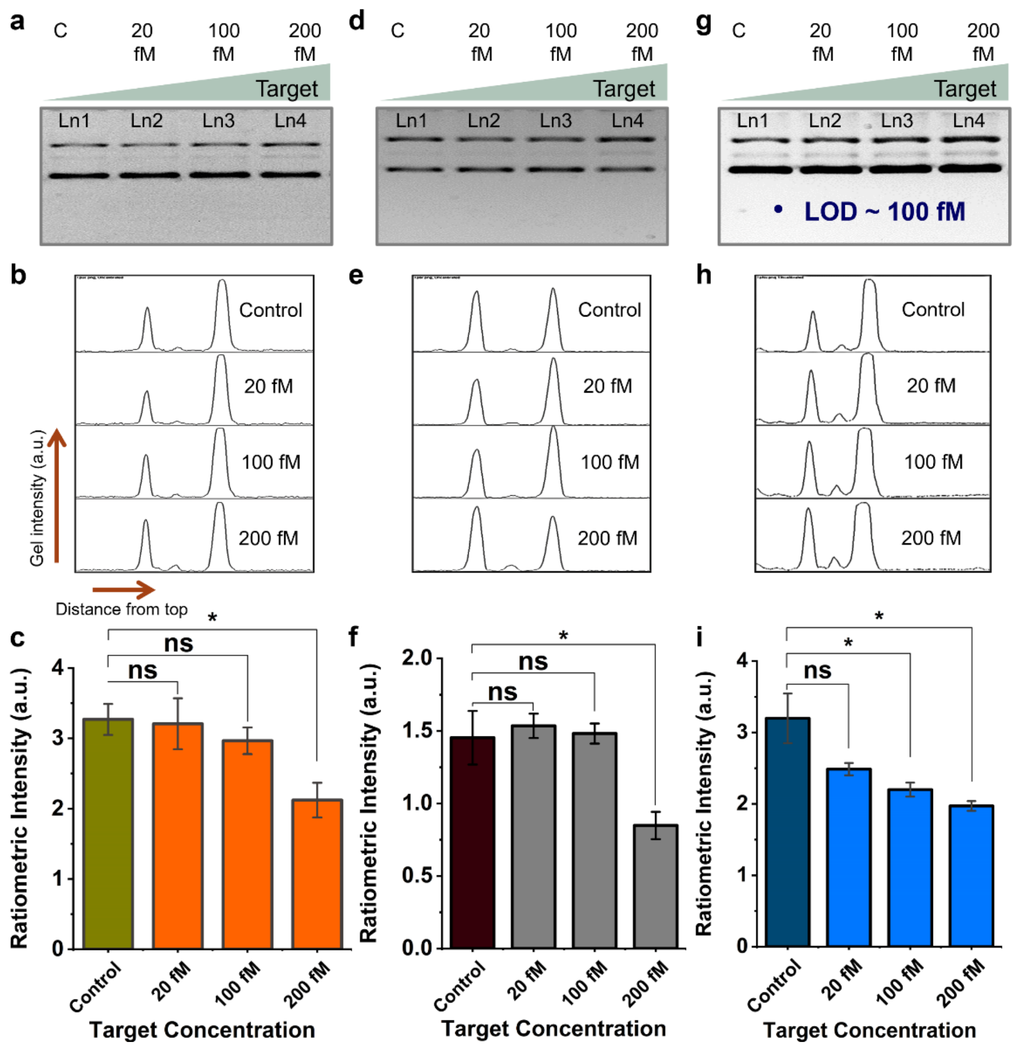

Supplementary Figure 7. Limit of Detection (LOD) determination of various plasmid reporters with synthetic ssDNA target (Target ssDNA_1, see the sequence in Tabe S1). Gel electrophoresis (1% agarose gel and 1×TBE buffer) demonstrating the CRISPR-Cas12a induced supercoil relaxation for various target concentrations using pUC19 (a), pBR322 (d), and ΦX174 (g). Gel intensity diagrams of each lane for pUC19 (b), pBR322 (e), and ΦX174 (h) reporters. Ratiometric intensity plotted in bar chart against different target concentrations for pUC19 (c), pBR322 (f), and ΦX174 (i) reporting systems. The assays were performed at 52 ˚C for 1 hour. Error bar represents the standard deviation of n=3 repeated experiments for each measurement. The graph shows statistical insignificance at $p>0.05$ (ns), and statistical significance at $p<0.05$ (*). Abbreviations: c, negative control; fM, femtomolar; Ln, lane; TBE, Tris-borate EDTA; a.u., arbitrary unit.

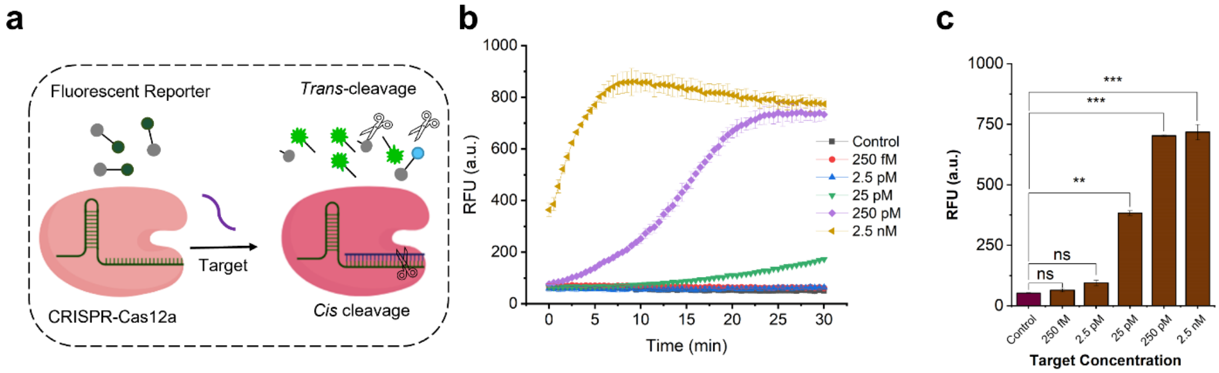

Supplementary Figure 8. CRISPR-LbCas12a-based detection using conventional fluorescent reporter molecules. (a) schematic diagram of CRISPR-LbCas12a assay. (b) RFU signal acquired for 30 min reaction with different target concentrations. (c) RFU reading taken after 1 hr of reaction against different target concentrations. Error bar represents the standard deviation of n=3 repeated experiments for each measurement. The graph shows statistical insignificance at p>0.05 (ns); and statistical significance at p<0.01 (**), and p<0.001(***). Abbreviations: RFU, relative fluorescence unit, ns, not significant.

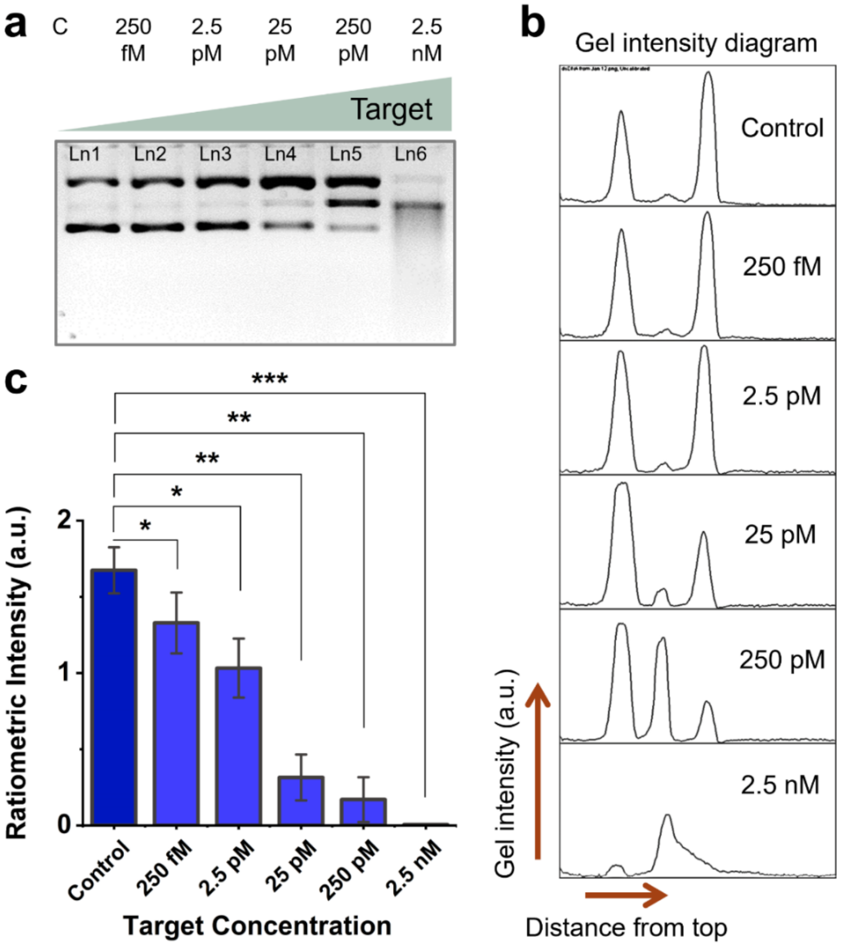

Supplementary Figure 9. Validation of rCRISPR for detecting dsDNA targets. (a) Gel electrophoresis (1% agarose gel and 1×TBE buffer) results demonstrating the nonspecific supercoil relaxation of ΦX174 DNA. Ln1: negative control (no target); Ln2, Ln3, Ln4, LN5, and Ln6: experimental lanes with target concentrations of 250 fM, 2.5 pM, 25 pM, 250 pM, and 2.5 nM, respectively. (b) Gel band intensity diagram. (c) Intensity plot against different concentrations of dsDNA target. These assays were performed at 52 ˚C for 1 hr. Error bar represents the standard deviation of n=3 repeated experiments for each measurement. The graph shows statistical significance at p<0.01 (*), p<0.01 (**), and p<0.001(***). Abbreviations: c, negative control; fM, femtomolar; pM, picomolar; nM, nanomolar; Ln, lane; TBE, Tris-borate EDTA.

2. Supplementary Tables

Supplementary Table 1. Oligos used for the study.

Item	Sequence (5’-3')
gRNA	UAAUUUCUACUAAGUGUAGAUCGUCGCCGUCCAGCUCGACC
Target ssDNA	GGT CGA GCT GGA CGG CGA CG
Target dsDNA	Sense strand: GGT CGA GCT GGA CGG CGA CGT AAA TCG ACG ACG CTG ACG GTA GCG AAT CGA TCG TAC GCT AGT CCG TAA TGT GAG TTG GCT GAT GGT TA<br>Antisense strand: TAA CCA TCA GCC AAC TCA CAT TAC GGA CTA GCG TAC GAT CGA TTC GCT ACC GTC AGC GTC GTC GAT TTA CGT CGC CGT CCA GCT CGA CC
gRNA_1	UAAUUUCUACUAAGUGUAGAUGUGCAAAUCUAUGCAAAACUG
Target ssDNA_1	TCA GTT TTG CAT AGA TTT GCA CA
gRNA_AAV	UA AUU UCU ACU AAG UGU AGA UCU CCA UCA CUA GGG GUUCCU
Target AAV	AGG AAC CCC TAG TGA TGG AG
gRNA_HPV16	UAA UUU CUA CUC UUG UAG AUU GAA GUA GAU AUG GCAGCAC
Target HPV16	GTG CTG CCA TAT CTA CTT CA
F-Q reporter	/6-FAM/ AAAAA /Dabcyl/

Supplementary Table 2. Performance comparison of various reporters.

Reporting types		LOD	Relative reaction rate	Easy of signal quantification	Relative Cost	Ref.
F-Q ssDNA reporter	**F**-TTATT-**Q**	~10-200 pM	High	High	High	[1,2]
	**F**-TTATT-5C-**Q**	20 pM	High	High	High	[2]
	**F**-8C-**Q**	-	Higher (K_{cat}= 0.43 per sec)	High	High	[3]
	**F**-10C-**Q**	-	Higher (higher background)	High	High	[3]
SPR ssDNA reporter	MOPCS	~15 fM	High	Easy	-	[4]
	CRISPR-SPR-FT	~5 aM	High	Easy	-	[5]
F-Q dsDNA reporter (<30bp)		~10 pM	Low	Easy	High	[6]
Long dsDNA reporter (sizing-based detection)		~0.25 pM	High	Hard	Low	[7]
Hybrid reporter		~0.25 pM	Low	Easy	Low	[8]
DNA supercoil relaxation		~100 fM	High	Easy	Low	This research

F: *Fluorescent molecule*

Q: *Quencher molecule*

Supplementary References:

1 Chen, J. S. *et al.* CRISPR-Cas12a target binding unleashes indiscriminate single-stranded DNase activity. *Science* **360**, 436-439, doi:10.1126/science.aar6245 (2018).

2 Lee, S. *et al.* Highly Efficient DNA Reporter for CRISPR/Cas12a-Based Specific and Sensitive Biosensor. *Biochip J.*, doi:10.1007/s13206-022-00081-0 (2022).

3 Lv, H. *et al.* Definition of CRISPR Cas12a trans-cleavage units to facilitate CRISPR diagnostics. *Front. Microbiol.* **12**, 766464 (2021).

4 Chen, Z. *et al.* A CRISPR/Cas12a-empowered surface plasmon resonance platform for rapid and specific diagnosis of the Omicron variant of SARS-CoV-2. *National Science Review* **9**, nwac104 (2022).

5 Chen, Y. *et al.* Ultrasensitive and specific clustered regularly interspaced short palindromic repeats empowered a plasmonic fiber tip system for amplification-free monkeypox virus detection and genotyping. *ACS nano* **17**, 12903-12914 (2023).

6 Smith, C. W. *et al.* Probing CRISPR-Cas12a Nuclease Activity Using Double-Stranded DNA-Templated Fluorescent Substrates. *Biochem.* **59**, 1474-1481, doi:10.1021/acs.biochem.0c00140 (2020).

7 Mohammad, N., Katkam, S. S. & Wei, Q. A Sensitive and Nonoptical CRISPR Detection Mechanism by Sizing Double-Stranded λ DNA Reporter. *Angew. Chem. Int. Ed.* **61**, e202213920, doi:https://doi.org/10.1002/anie.202213920 (2022).

8 Mohammad, N., Talton, L., Hetzler, Z., Gongireddy, M. & Wei, Q. Unidirectional trans-cleaving behavior of CRISPR-Cas12a unlocks for an ultrasensitive assay using hybrid DNA reporters containing a 3′ toehold. *Nucleic Acids Res.* **51**, 9894-9904 (2023).
